# Transient elastography score is elevated during rheumatoid factor-positive chronic hepatitis C virus infection and rheumatoid factor decline is highly variable over the course of direct-acting antiviral therapy

**DOI:** 10.1371/journal.pone.0267512

**Published:** 2022-04-28

**Authors:** Ann W. N. Auma, Corinne Kowal, Carey L. Shive, Alyssa Lange, Sofi Damjanovska, Elizabeth Zebrowski, Elane Reyes, Leonard Calabrese, Lenche Kostadinova, Yngve Falck-Ytter, Maya Mattar, Donald D. Anthony

**Affiliations:** 1 Department of Pathology, Department of Medicine Case Western Reserve University, Cleveland, Ohio, United States of America; 2 Cleveland VA Medical Center and VA GRECC, Cleveland, Ohio, United States of America; 3 Department of Rheumatologic and Immunologic Disease, Cleveland Clinic Foundation, Cleveland, Ohio, United States of America; 4 Division of Rheumatology, MetroHealth Medical Center, Cleveland, Ohio, United States of America; Harvard Medical School, UNITED STATES

## Abstract

**Background:**

Elevated rheumatoid factor (RF) levels and systemic immune activation are highly prevalent during chronic hepatitis C virus (HCV) infection. Direct-acting antiviral (DAA) therapy has been associated with normalization of various soluble immune activation parameters. Whether the RF levels relate to soluble immune activation markers during chronic HCV infection, and over what time frame RF levels normalize during and after DAA treatment is unknown and was investigated here.

**Methods:**

In a longitudinal study, plasma and serum was obtained from HCV infected RF positive (RF+) and RF negative (RF-) participants. The levels of RF, HCV RNA and soluble markers of inflammation were determined before (week 0), during (weeks 4, 8 and 12) and after (week 24) treatment with HCV DAA therapy. In a subset of RF+ participants, the analysis was extended to over 70 weeks after therapy initiation. Hepatic and other clinical parameters were determined at baseline (week 0) in all participants.

**Results:**

Before therapy, transient elastography (TE) score was greater in RF+ compared to RF- HCV infected participants, while the systemic levels of soluble inflammatory markers were comparable. Following DAA therapy initiation, HCV RNA levels became undetectable within 4 weeks in both the RF+ and RF- groups. RF levels declined in the first 6 months in most RF+ persons but most commonly remained positive. The levels of some soluble inflammatory markers declined, mainly within 4 weeks of DAA therapy start, in both the RF+ and RF- groups. The baseline (week 0) TE score correlated with RF levels before, during and after DAA therapy, while plasma IL-18 levels correlated with RF level after DAA therapy.

**Conclusion:**

During chronic HCV infection, TE score is elevated in RF+ HCV infected individuals and factors other than HCV viremia (including liver stiffness or fibrosis and select markers of inflammation) likely contribute to persistence of RF after treatment of HCV with DAA.

## Introduction

In patients with hepatitis C virus (HCV) infection, extrahepatic manifestations like arthralgia are frequent, as are elevated serum levels of rheumatoid factor (RF) [1]. RFs are immunoglobulin (Ig) M autoantibodies that bind to the Fc portion of IgG are capable of forming immune complexes that can contribute to the pathogenesis of autoimmune disease [2]. The percentage of HCV infected individuals with elevated RF levels ranges from 30% to 80% and IgM RFs are often a component of cryoglobulins [3, 4]. Cryoglobulins are cold-perceptible proteins composed of a mixture of monoclonal or polyclonal IgM with RF activity and polyclonal IgG bound to HCV that form immune complexes. HCV infection is the leading cause of mixed cryoglobulinemia (MC), accounting for 85–95% of all cases [5, 6]. MC is often detected in 25% to 30% of chronic HCV infected individuals and the syndrome of MC with vasculitis is observed in a smaller subset of these persons, characterized by the deposition of RF-containing immune complexes in the vascular endothelium in organs such as the skin, kidneys, and peripheral nerves [7–10]. Although causal mechanisms underlying the formation of cryoglobulins and vasculitis are somewhat unclear, HCV viremia is the predominant underlying etiological factor and RF is a central feature of the pathogenic immune complexes [11]. Thus, understanding the direct and indirect contributions of HCV viremia to elevated RF levels could help guide strategies to manage patients with vasculitis during and after DAA therapy [12]. Given that DAA therapy targets HCV replication and has been associated with a rapid >95% sustained virologic response (SVR), we investigated the RF level kinetics during DAA therapy, and how this relates to select inflammatory parameters involved in the pathogenesis of hepatic inflammation.

## Materials and methods

### Study population

Study participants provided written informed consent under protocols approved by the institutional review boards for human studies at the Cleveland Veterans Affairs Medical Center. In a longitudinal study, RF positive (RF+) persons (RF>24 IU/mL) with concurrent chronic HCV infection were sampled before (week 0, n = 44), during (week 4, n = 36; week 8, n = 30) and after (week 24, n = 26) the initiation of HCV DAA therapy. Laboratory analysis was performed at all time points where sample availability permitted. A subset of the participants (n = 15) was followed up over 70 weeks (range of 74–158 weeks) after therapy start. RF intermediate persons (RF = 17–24 IU/mL, n = 6) and RF negative (RF-) (RF<16 IU/mL, n = 10) with concurrent chronic HCV infection were followed longitudinally at the same time points; before, during and after HCV DAA therapy. Chronic HCV infected persons were positive for HCV antibody and had detectable HCV RNA for >6 months, and were negative for HIV (Human Immunodeficiency Virus) by Enzyme-Linked ImmunoSorbent Assay (ELISA) and HBV (Hepatitis B Virus) with HBsAg negative and HBcAb negative [6]. Duration of HCV infection was estimated based on the date of reported risk factors for HCV infection during HCV clinic intake evaluation. All participants underwent DAA therapy (Sofosbuvir/Ledipasvir, n = 45; Ombitasvir/Paritaprevir/Ritonavir, n = 1; Dasabuvir/Ombitasvir/Paritaprevir/Ritonavir, n = 1; or Sofosbuvir/Semeprivir, n = 3) for a period 8–12 weeks as per standard of care (8 weeks for persons with TE values < 8.5 kPa and 12 weeks for persons with TE values > 8.5 kPa).

### Clinical laboratory and radiology investigations

Certified clinical laboratories performed investigations of aspartate transaminase (AST), alanine transaminase (ALT), platelet count, and albumin level. Fibrosis 4 index (Fib-4) score, and AST to platelet ratio index (APRI) were calculated. Liver stiffness measurement was performed using transient elastography (TE; TE model 502, Echosens, France).

### ELISA

Plasma from the study participants was assessed at week 0 (before), during (weeks 4, 8 and 12) and after (weeks 24 and 70+) treatment with HCV DAA therapy for inflammatory markers including soluble cluster of differentiation 14 (sCD14; R&D Systems), interferon gamma-inducible protein 10 (IP10; R&D Systems), autotaxin (ATX; R&D Systems), Mac-2-binding protein (Mac-2BP; Invitrogen, Thermo Fisher Scientific), human C reactive protein (CRP; R&D Systems), and interleukin 6 (IL-6; R&D Systems) by ELISA. Serum was assessed for IgM RF (ALPCO) by ELISA at time points; weeks 0, 4, 8, 12, 24 and 70+. RF ranges were described in the IgM RF ELISA kit (ALPCO)).

### Statistical analysis

Differences between two study groups were determined by Mann Whitney test and between two timepoints by Wilcoxon and Mann Whitney tests. Associations between continuous variables per group were evaluated using Spearman’s rank correlation coefficient. Tests were performed in GraphPad Prism, version 8 and SPSS version 27. P values <0.05 were considered statistically significant for all tests.

## Results

### Study participant characteristics

The RF+ (n = 44) and RF- (n = 10) groups are shown in Table 1. The TE score (obtained prior to HCV therapy) was higher in the RF+ group when compared to the RF- group (median 8.7 kPa vs 5.8 kPa, p = 0.04) while the other clinical hepatic parameters (AST, ALT, albumin level, Fib-4, APRI and platelet count) were comparable. HCV genotype 1A was predominant in both the RF+ (62%) and RF- (70%) groups. Hepatocellular carcinoma (HCC, 11%) and mortality (16%) was observed over the duration of longitudinal sampling only in the RF+ group and not in the RF- group. Rheumatoid arthritis was not observed in either the RF+ or RF- groups.

**Table 1:** Clinical characteristics of HCV infected RF+ and RF- participants at baseline (before the initiation of DAA therapy).

	RF+	RF-	P value
	n = 44	n = 10	RF+ vs RF-
**Age, years**	60 (56; 64)	58 (56;62)	0.46
**Gender; No. (%)**			
**Male**	43 (98%)	10 (100%)	
**Female**	1 (2%)	0	
**Race; No. (%)**			
**Black**	27 (61%)	6 (60%)	
**White**	15 (34%)	4 (40%)	
**Other**	2 (5%)	--	
**Albumin level (g/dL)**	3.7 (3.5; 3.8)	3.6 (3.5; 3.9)	0.71
**ALT level (U/L)**	58 (36; 90)	46 (31; 84)	0.35
**AST level (U/L)**	50 (31; 86)	37 (28; 66)	0.29
**Platelets (x10^9/L)**	184 (149; 243)	221 (183; 276)	0.14
**ALC**	2.1 (1.8; 2.6)	2.2 (2.0; 2.4)	0.62
**Rheumatoid Factor (IU/mL)**	51 (36; 235)	4 (2; 7)	<.0001*
**APRI; No. (%)**	n = 42	n = 10	
**<0.4**	11 (26%)	4 (40%)	
**0.4–1.5**	20 (48%)	5 (50%)	
**>1.5**	11 (26%)	1 (10%)	
**Fibrosis 4 index**	2.0 (1.3; 3.6)	1.2 (1.0; 2.6)	0.15
**HCV Genotype; No. (%)**			
**1a**	27 (62%)	7 (70%)	
**1b**	13 (30%)	3 (30%)	
**2b**	1 (2%)	--	
**3**	1 (2%)	--	
**4b**	1 (2%)	--	
**Unknown**	1 (2%)	--	
**Fibroscan Score**	8.7 (6.1; 11.5)	5.75 (5.0; 7.2)	0.04*
**Hypertension**	33 (75%)	6 (60%)	
**Statin Use**	25 (57%)	3 (30%)	
**Diabetes Mellitus**	18 (40%)	2 (20%)	
**Hepatocellular Carcinoma Diagnosis**	5 (11%)	--	
**BMI**	29.3 (26.5; 31.4)	33.65 (28.8; 34.8)	0.07
**Mortality**	7 (16%)	--	

Median (25^th^, 75^th^ percentiles) shown unless otherwise indicated

[No. (%)]

*Statistically significant (P value <0.05) using Mann Whitney test for unpaired comparison

Abbreviations:

ALT, alanine aminotransferase;

APRI, aspartate aminotransferase to platelet ratio index;

AST, aspartate aminotransferase;

ALC, Absolute lymphocyte count

BMI, Body Mass Index

HCV, hepatitis C virus.

Fibrosis 4 index calculated as [age × AST level] / [platelet count ×√ALT level].

### RF levels decline but frequently remain positive during and after HCV DAA therapy, and TE scores are higher in RF+ compared to RF- subgroups prior to HCV therapy

**Fig 1:**
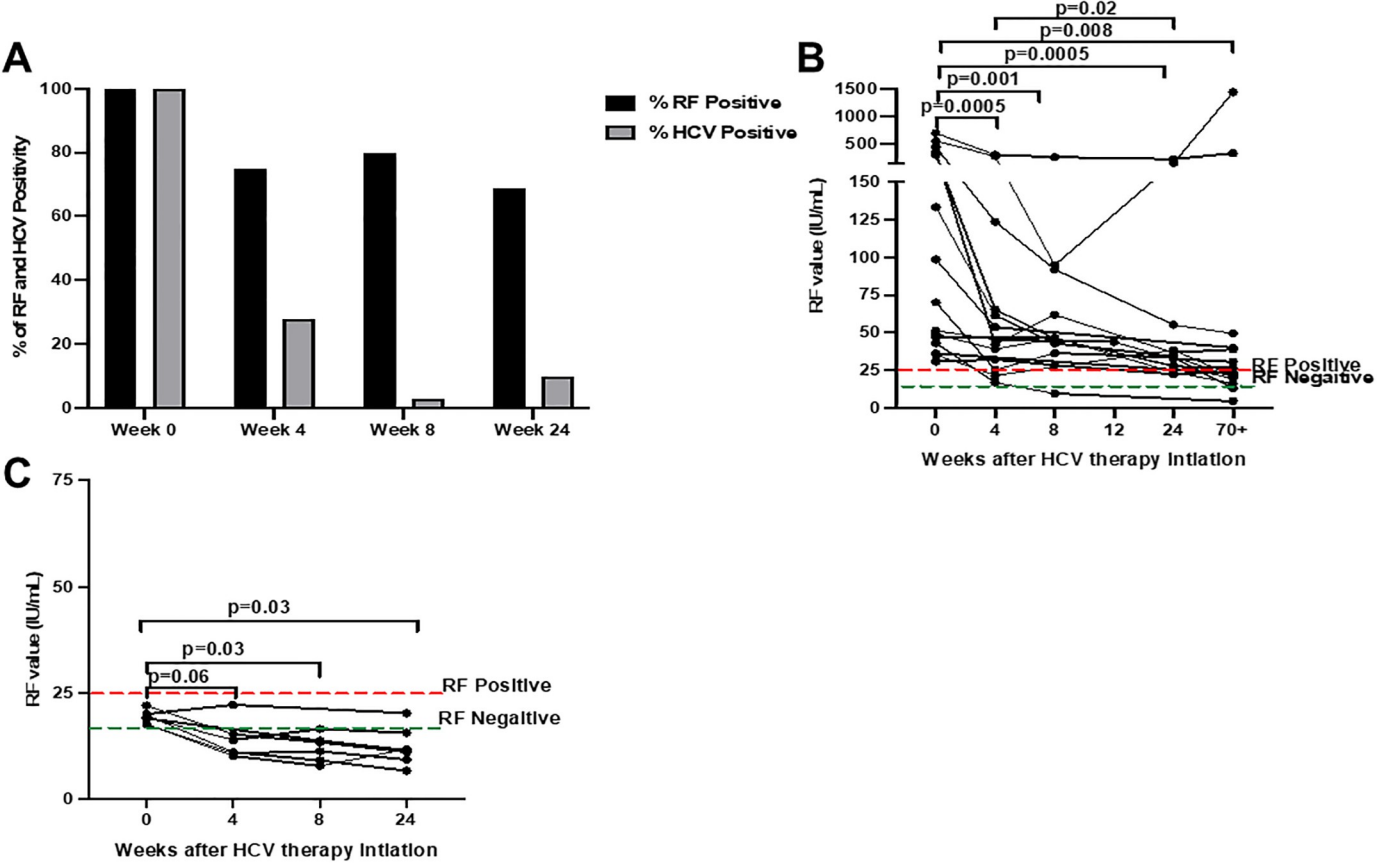
Rheumatoid factor (RF) positivity commonly persists in hepatitis C virus (HCV) infected persons during and after the course of direct-acting antiviral (DAA) therapy. HCV infected (HCV+) RF positive (RF+) persons were followed longitudinally over the course of HCV DAA therapy; before (week = 0: n = 44), during (week 4: RF+ n = 27/36, 75%; HCV+ n = 11/39, 28%), (week 8: RF+ n = 24/30, 80%; HCV+ n = 1/39, 3%) and after (week 24: RF+ n = 18/26, 69%; HCV+ n = 4/39, 10%) treatment, where the proportion of the persons that were RF+ (>24 IU/mL) and had detectable serum HCV levels was determined **(A)**. A subset of this group (week 0, n = 15) was followed over an additional extended time frame after HCV DAA therapy initiation at week 4: RF+ n = 11/13, 85%; week 8: RF+ n = 10/11, 91%; week 12: RF+ n = 1/1, 100%; week 24: RF+ n = 11/13 85%; and at least 70 weeks out (week 74–158): RF+ n = 7/15 47%; and the serum RF values determined at each timepoint **(B)**. RF intermediate (n = 6, 17–24 IU/mL) **(C)** persons that were HCV+ was followed up and the serum RF values determined at each timepoint along the course of HCV DAA therapy. Differences between timepoints were determined by the paired Wilcoxon test.

We investigated first whether DAA-induced eradication of HCV viremia was associated with rapid or delayed normalization of RF levels. RF IgM levels >24 IU/mL and <16 IU/mL were used to define RF+ and RF- respectively while 17–24 IU/mL levels were considered intermediate. Following the initiation of DAA therapy in the RF+ HCV infected persons, a high proportion of this group consistently had detectable RF levels during (week 4: n = 27/36, 75% and week 8: n = 24/30, 80%) and after (week 24: n = 18/26, 69%) treatment (Fig 1A). As expected, DAA treatment was associated with a dramatic decline in HCV RNA levels in almost all RF+ persons soon after therapy initiation (week = 0: n = 44/44, 100% HCV RNA+; week 4: n = 11/39, 28%; week 8: n = 1/39, 3% HCV RNA detectible) (Fig 1A). Further, all but 4 persons attained SVR (undetectable HCV RNA at 12 weeks after therapy completion), or 90%. Failure of achieving SVR in these 4 persons was attributed to poor adherence to therapy (medication non-compliance, discontinuation of medications due to adverse effects) or re-exposure to HCV (n = 1) (Fig 1A). In the RF- group, serum HCV RNA became undetectable in all persons (n = 10/10, 100%) within 4 weeks of DAA therapy, a response that was sustained after treatment completion (100% SVR at 24 weeks).

In a subset of RF+ HCV infected persons, (n = 15), RF levels were measured longitudinally up to and beyond 70 weeks after therapy initiation. In this subgroup, the RF levels declined within 4 weeks of therapy initiation (weeks 0 vs 4: 70.1 vs 45.2 IU/mL, p = 0.0005), while the proportion of the RF+ group that remained RF+ was persistently high at all timepoints during and after DAA therapy (week 4: 11/13, 85% RF+; week 8: 10/11, 91% RF+; week 12: 1/1, 100% RF+; week 24: 11/13 RF+, 85% and >70 weeks: 7/15, 47% RF+) (Fig 1B). We also longitudinally analyzed the RF levels in the RF intermediate and RF- persons before, during and after therapy. In the RF intermediate group, the RF levels declined during (weeks 0 vs 8: 19.3 vs 12.4 IU/mL, p = 0.03) and after (weeks 0 vs 24: 19.3 vs 11.5 IU/mL, p = 0.03) DAA therapy, with the majority becoming RF- (week 4: n = 5/6, 83%; week 8: n = 6/6, 100%; week 24: n = 6/7, 86%) (Fig 1C). In the RF- group, there were no statistically significant changes in RF levels and all persons remained RF- during and after therapy (S1 Fig). We also examined whether duration of HCV infection was associated with RF positivity and/or levels. The estimated duration of HCV infection was comparable between the RF+ and RF- groups (RF- vs RF+: 35 vs 40 years, p = 0.16; not shown). Additionally, we did not find any correlations between the duration of HCV infection and the RF levels before, during or after DAA therapy in the RF+ and RF- groups (not shown).

Overall, although effective DAA treatment of chronic HCV infection was associated with a decline in RF levels in the RF+ group, RF levels were persistently detected in the majority of patients over a 6-month time window, and remained positive over 70 weeks after start of DAA therapy in a substantial portion as well.

### RF positivity is associated with TE score before and after HCV DAA therapy

**Fig 2:**
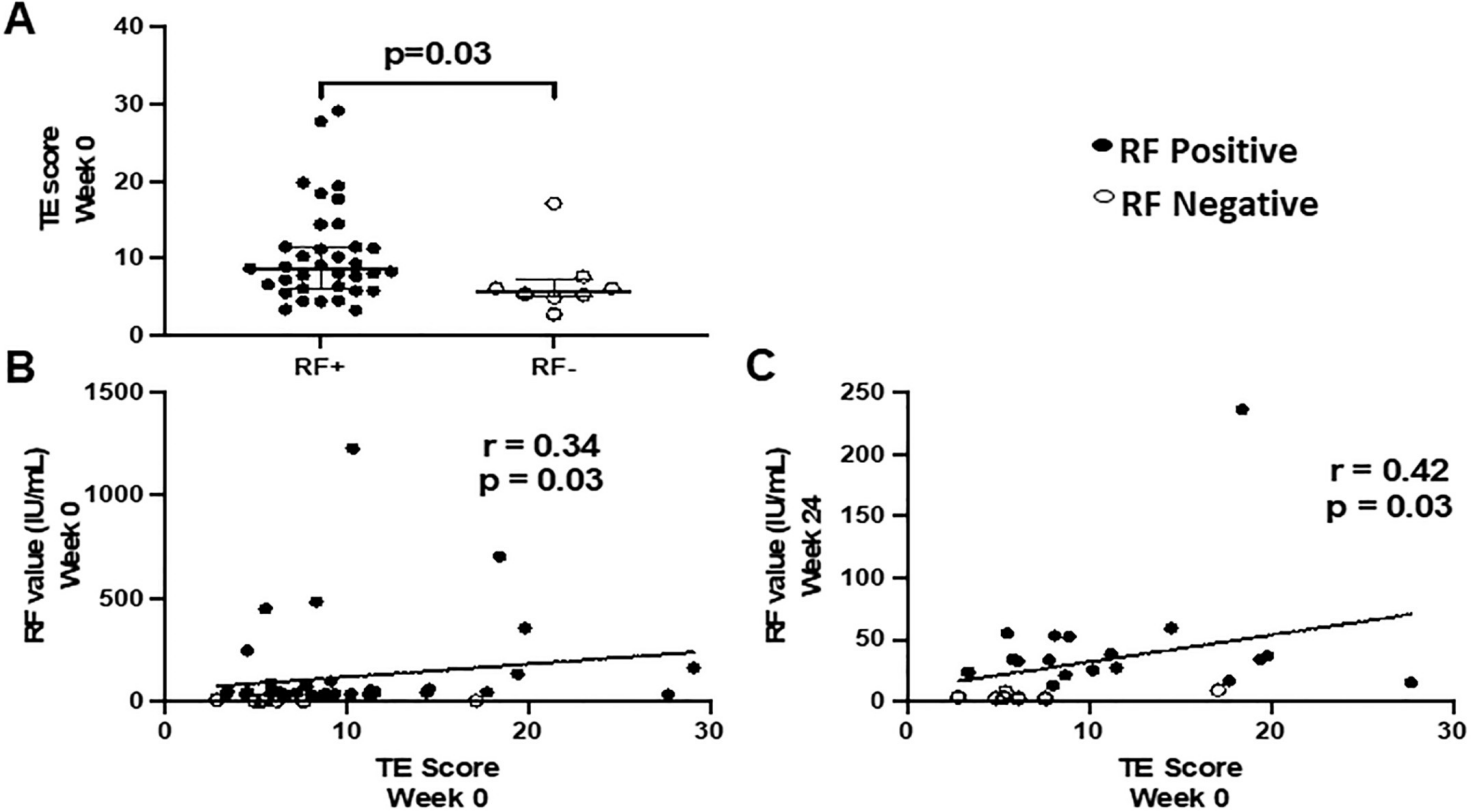
TE score is elevated in rheumatoid factor (RF) positive persons and the baseline TE score correlates with RF values over the course of hepatitis C virus (HCV) direct-acting antiviral (DAA) therapy. Panel A: TE scores prior to DAA therapy are shown for RF+ (n = 44) and RF- (n = 8) HCV infected persons. Correlations between baseline TE score and RF level at weeks 0 (panel B) and week 24 (panel C) are shown. Mann-Whitney test was used for comparison between two groups. Spearman’s Rho correlation was used to determine correlations between two values.

The prevalence of MC increases with the duration of the hepatic illness and chronic liver disease, especially due to HCV infection [13–16]. Serum cryoglobulinemia is also associated with liver fibrosis [14], so there is an established relation between liver disease and RF. Here, we focused on the relation between select parameters of inflammation during HCV infection, TE score, and the rapid time window of HCV clearance during DAA therapy to understand potential driving factors of RF positivity (direct effect of virus vs. indirect effects attributable to hepatic and/or systemic inflammation). TE scores measure liver stiffness, a combination of liver inflammation and scarring (fibrosis) [17, 18]. RF+ persons displayed higher TE scores compared to RF- HCV infected persons (Table 1 and Fig 2). Additionally, the baseline (week 0) TE score positively correlated with the RF levels before (week 0: r = 0.34, p = 0.03, n = 43; Fig 2B), during (week 4: r = 0.36, p = 0.03, n = 36; week 8: r = 0.41, p = 0.02, n = 31; not shown) and after (week 24: r = 0.42, p = 0.03, n = 27; Fig 2C) DAA therapy in the RF+ and RF- groups combined. However, in the smaller RF+ and RF- subgroups, TE scores did not correlate with the RF levels (not shown).

Furthermore, RF levels before DAA therapy correlated with platelet count and APRI (RF week 0 vs platelet count week 0: r = -0.30, p = 0.05, n = 44; RF week 0 vs APRI week 0: r = 0.63, p = 0.02, n = 14; not shown). RF levels during DAA therapy correlated with albumin level RF week 4 vs albumin level week 4: r = -0.42, p = 0.03, n = 26; not shown). RF levels after DAA therapy completion did not correlate with liver function parameters (AST, ALT, albumin, Fib-4 or APRI). TE scores before DAA therapy correlated with AST, ALT, platelet count and APRI (TE week 0 vs AST week 0: r = 0.42, p = 0.01, n = 34; TE week 0 vs ALT week 0: r = 0.50, p = 0.003, n = 34; TE week 0 vs platelet count week 0: r = -0.38, p = 0.03, n = 35; TE week 0 vs APRI week 0: r = 0.45, p = 0.007, n = 34; not shown).

### TE score positively correlates with plasma IL-18 levels during therapy and RF levels post-therapy completion in RF+ persons

Chronic HCV infection is associated with elevated plasma levels of systemic markers of immune activation such as sCD14, sCD163, IL-18, Mac-2BP, ATX, and IP-10, more so in patients with advanced liver disease [19–22]. The levels of some markers normalize following DAA therapy initiation (such as IP-10) while other markers often remain elevated (such as sCD14) [19, 23, 24]. Residual immune activation following DAA therapy has been attributed to presence of liver fibrosis and cirrhosis prior to DAA treatment [23, 24]. In the current study, DAA treatment in the RF+ group (particularly within 4 weeks of therapy) was associated with a decline in levels of ATX (p = 0.05), IP-10 (p = 0.008), Mac-2BP (p = 0.003), sCD163 (p = 0.0003), and IL-18 (p = 0.03), and an increase in CRP levels (p = 0.05), while IL-6 and sCD163 levels remained unchanged (S2 Fig). We next determined whether baseline TE score (week 0, before therapy) was associated with levels of inflammatory markers before (week 0), during (week 4 and 8) or after (week 24 and 70+) DAA therapy in the RF+ group. Before therapy (week 0), we observed a positive correlation between TE score and the levels of sCD14 (r = 0.68, p = 0.03), ATX (r = 0.70, p = 0.04) and Mac-2BP (r = 0.77, p = 0.01) (S1 Table). Baseline TE score also positively correlated with levels of IL-18 (week 4: r = 0.90, p = 0.005), sCD14 (week 4: r = 0.75, p = 0.03; week 8: r = 0.79, p = 0.03) and Mac-2BP (week 8: r = 0.88, p = 0.007) on therapy (S1 Table). Baseline TE score also positively correlated with levels of Mac-2BP after therapy completion (week 70+: r = 0.60, p = 0.04) (S1 Table).

We then investigated a potential mechanistic linkage between RF levels and the levels of these inflammatory markers before (week 0), during (week 4 and 8) or after (week 24 and 70+) DAA therapy by evaluating correlations between these parameters at each time point in the RF+ group (S2 Table). In the RF+ group, there were no correlations between plasma levels of inflammatory markers and RF levels before therapy (week 0; not shown). During therapy, RF levels (week 8) negatively correlated with sCD14 levels (week 70+; r = -0.70, p = 0.01) (S2 Table). After therapy, the RF levels (week 24) positively correlated with IL-18 levels (week 24; r = 0.72, p = 0.02) (S2 Table), as well as IL-18 levels at week 0 (r = 0.62, p = 0.03). Together, IL-18 levels were positively associated with TE scores before, during DAA treatment and with RF levels following DAA treatment completion in RF+ persons.

## Discussion

We investigated the impact of HCV DAA therapy on the RF levels in RF+ (>24 IU/mL) and RF- (<16 IU/mL) persons with chronic HCV infection and explored the relationship between RF levels and hepatic functional parameters, as well as soluble immune activation markers and mediators. Following the initiation of DAA therapy, the RF levels rapidly declined within the first 4 weeks and continued to decline up to 70 weeks after initiation of therapy in the RF+ group. Despite this decline, RF levels persistently remained positive in most patients in the RF+ group in the short-term (85% at 4 weeks) and long-term (47% at or beyond 70 weeks). Similarly, RF levels declined in the RF intermediate group following DAA therapy initiation. In the RF- group, the RF levels generally remained unchanged and were consistently negative regardless of DAA therapy. TE scores were higher in the RF+ group when compared to the RF- group prior to therapy, and TE score correlated with RF level at multiple time points. Plasma levels of IL-18 correlated with RF levels in the RF+ group after DAA therapy.

Chronic HCV infection is associated with elevated RF levels [3, 4]. The immune response to HCV may induce the development of RF, cryoglobulins and other autoimmune related mediators [4]. Presence of RF has been associated with more severe disease forms of rheumatoid arthritis [25, 26]. Notably, here in the RF+ group, but not RF- group, we observed mortality and the development HCC, suggesting that RF could be one predictor of poor disease outcome in chronic HCV infected patients. Published literature on the impact of DAA therapy on RF is quite limited [27]. Moreover, there are conflicting reports on the changes in the levels of circulating cryoglobulins (containing IgM RF) after effective DAA treatment and attainment of SVR [28, 29]. Here, we report a gradual decline in the RF levels as early as 4 weeks after initiation of DAA therapy, but levels often persist up to at least 70 weeks, demonstrating the longevity of immune derangement in the RF+ persons. Our finding slightly differs from a previous report that found a significant reduction of RF levels only during but not after DAA therapy at time of SVR measurement (12 weeks after completion of therapy) [27]. Interestingly, we saw the most pronounced decline in RF levels and distinct clearance of plasma HCV RNA occurring within the same time frame; 4–8 weeks after initiation of therapy. This data supports previous reports that claim HCV itself is a major, perhaps direct, etiological agent driving the production of the autoantibodies contained in cryoglobulins (including IgM RF) [30–35]. However, there appears to be a delayed and likely indirect relationship in a substantial number of individuals.

Indirect causes of IgM RF and cryoglobulin production may include 1) HCV-mediated hepatic edema and scarring or cirrhosis and the consequent immunologic milleu and 2) the action of extrahepatic inflammatory immunocyte activating mediators which are secreted during chronic HCV infection. First, the impairment of liver parenchyma integrity and function has been implicated in the production of circulating cryoglobulins (include IgM RF) [36, 37]. Along this line, we showed that TE score was elevated in the RF+ group when compared to the RF- group. Further, our findings demonstrating a correlation between plasma RF levels and TE scores extend previous reports of a positive association between circulating cryoglobulin levels and advanced liver fibrosis [14]. This suggests that inadequate restoration of liver structure and function after successful DAA therapy could continue to contribute to RF persistence as supported by evidence of associations of RF levels and TE scores with the poor hepatic synthetic function (determined by albumin levels and platelet counts) and hepatic inflammation and fibrosis (depicted by AST, ALT, APRI and Fib-4) before, during and after DAA therapy. Our findings of a positive correlation between TE scores and the levels of IL-18, CD14 and Mac-2BP during and after effective DAA therapy may support a mechanistic link between immune activation mediators and residual liver damage, however, this data should be interpreted with caution due to limitations in sample size across the various timepoints. Mac-2BP glycoprotein is a biomarker of liver fibrosis produced by Kupffer cells and CD14 is a glycoprotein that is a co-receptor for recognition of lipopolysaccharide and is shed from the surface of liver-resident and circulating activated macrophages and monocytes. Soluble CD14, IL-18 and other immune activation mediators are produced in abundance in the liver in response to HCV and can act to perpetuate intrahepatic inflammation and progression of fibrosis and cirrhosis of the liver [38, 39]. During hepatic fibrosis and cirrhosis, increased hepatic portal pressure and/or gut permeability may cause microbial translocation, directly resulting in hepatic inflammation and subsequent production of IgM RF given the current and previous evidence of elevated peripheral levels of CD14 and LPS respectively during chronic HCV infection [40]. Alternatively, the impaired filtration function of the liver can indirectly contribute to accumulation of cryoglobulin immune complexes that are composed of IgM RF among other proteins [16]. The duration of liver disease may also be a contributory factor in the production of RF-containing cryoglobulins since patients with cryoglobinemia often have a longer history of hepatitis (regardless of etiology) [36]. However, duration of HCV infection was not associated with higher RF levels in our study groups.

Chronic HCV infection has been associated with enhanced production of systemic immune activation markers and mediators, and these factors may in part contribute to RF production. We observed RF levels to positively correlate with IL-18 levels in the RF+ group after DAA therapy completion (at the SVR time point). Given that this relationship became apparent after HCV RNA clearance, it is plausible that IL-18 is one of the cytokines indirectly contributing to RF production. IL-18 is a pro-inflammatory cytokine frequently elevated in chronic viral infections (such as HCV infection) and in liver disease (regardless of etiology) [41–43]. Moreover, there is evidence of enhanced IL-18 production (*in vitro* and *in vivo*) from studies of individuals with HCV-associated liver disease and concurrent cryoglobulinemia when compared to HCV infected controls with no cryoglobulinemia, suggesting that IL-18 and other proinflammatory cytokines may contribute to the presence of cryoglobulins during chronic HCV infection [44, 45]. IL-18 may exert a stimulatory effect on the IgM RF producing B cells since there is evidence that IL-18 can promote/induce B cell activity such as autoantibody production [46].

Limitations of the current study include a small sample size, lack of examination of TE scores after the initiation of DAA therapy, lack of examination of liver tissue for markers and potential mediators of inflammation and fibrosis, and inability to measure circulating cryoglobulin levels due to the lack of real-time immunologic sample analysis.

In summary, we have shown that although RF levels declined during and after DAA therapy in RF+ patients with chronic HCV infection, RF levels persisted in a large portion of these persons in spite of successful HCV DAA therapy. Further, the TE scores were elevated in the RF+ compared to RF- persons and the RF levels positively correlate with TE scores and specific immune activation mediators including IL-18 and sCD14. These findings suggest a heterogeneity in RF decline during HCV DAA therapy possibly due to indirect mechanisms that persist even after removal of HCV from the host.

## Supporting information

S1 Data (XLSX)

S1 Table Correlations between baseline (week 0) transient elastography score and levels of soluble markers of immune activation before, during and after HCV DAA therapy in RF+ persons. (DOCX)

S2 Table Correlations between levels of soluble markers of immune activation and RF levels before, during and after HCV DAA therapy in RF+ persons. (DOCX)

S1 Fig Rheumatoid factor (RF) levels are not altered by direct-acting antiviral (DAA) therapy in RF negative hepatitis C virus (HCV) infected persons. HCV infected (HCV+) RF negative (RF-; n = 10, <16 IU/mL) persons were followed longitudinally over the course of HCV DAA therapy and the serum RF values determined before (week = 0: n = 10), during (week 4: RF+ n = 0/10, 0%; HCV+ n = 0/10, 0%), (week 8: RF+ n = 0/10, 0%; HCV+ n = 0/10, 0%) and after (week 24: RF+ n = 0/10, 0%; HCV+ n = 0/10, 0%) treatment, where the proportion of the persons that were RF+ (>24 IU/mL) and had detectable serum HCV levels was determined. Differences between timepoints were determined by the paired Wilcoxon test.
(TIFF)

S2 Fig Plasma levels of systemic immune activation markers declined within the first 4 weeks of HCV DAA therapy in RF+ HCV infected persons. The plasma levels of systemic markers of immune activation including A) IP-10 B) MAC2-BP C) sCD163 D) autotaxin D) CRP E) sCD14 F) IL-6 and IL-18 were determined by ELISA at time points before (week 0), during (weeks 4, 8 and 12) and after (week 24) DAA therapy. Differences between time points were determined by the paired Wilcoxon test.
(TIF)
