# Supplementary material for: Transient elastography score is elevated during rheumatoid factor-positive chronic hepatitis C virus infection and rheumatoid factor decline is highly variable over the course of direct-acting antiviral therapy
Source: PLoS One. 2022 Apr 28;17(4):e0267512. doi: 10.1371/journal.pone.0267512 (PMC9049346; doi:10.1371/journal.pone.0267512)
Supplement: S1 Table — (DOCX) [file pone.0267512.s002.docx]

**S1 Table: Correlations between baseline (week 0) transient elastography score and levels of soluble markers of immune activation before, during and after HCV DAA therapy in RF+ persons**

|  | **Week 0** | **Week 4** | **Week 8** | **Week 70+** |
| --- | --- | --- | --- | --- |
| **Transient Elastography score at**  **Week 0** | **IL-18 (pg/mL),** n=9 | | | |
|  | r = 0.39,  p = 0.23 | r = 0.90,  p = 0.005* | r = 0.67,  p = 0.08 | r = 0.49,  p = 0.13 |
|  | **Soluble CD14 (ng/mL),** n=10 | | | |
|  | r = 0.68,  p = 0.03* | r = 0.75,  p = 0.03* | r = 0.79,  p = 0.03* | r = 0.01,  p = 0.99 |
|  | **MAC-2BP (ng/mL),** n=12 | | | |
|  | r = 0.77,  p = 0.01* | r = 0.53,  p = 0.15 | r = 0.88,  p = 0.007** | r = 0.60,  p = 0.04* |
|  | **Autotaxin (ng/mL),** n=9 | | | |
|  | r = 0.70,  p = 0.04* | r = 0.37,  p = 0.33 | r = 0.12,  p = 0.79 | r = 0.79,  p = 0.09 |

**Soluble markers are shown where significant correlations were observed at any time point.**

* Statistically significant (P value <0.05) using Spearman's rank sum test.
