# Supplementary material for: Transient elastography score is elevated during rheumatoid factor-positive chronic hepatitis C virus infection and rheumatoid factor decline is highly variable over the course of direct-acting antiviral therapy
Source: PLoS One. 2022 Apr 28;17(4):e0267512. doi: 10.1371/journal.pone.0267512 (PMC9049346; doi:10.1371/journal.pone.0267512)
Supplement: S2 Table — (DOCX) [file pone.0267512.s003.docx]

**S2 Table: Correlations between levels of soluble markers of immune activation and RF levels before, during and after HCV DAA therapy in RF+ persons**

|  | **RF levels (IU/mL) Week 0** | **RF levels (IU/mL)**  **Week 4** | **RF levels (IU/mL)**  **Week 8** | **RF levels (IU/mL)**  **Week 24** | **RF levels (IU/mL)**  **Week 70+** |
| --- | --- | --- | --- | --- | --- |
| **Soluble CD14 (ng/mL)**  **Week 70+** (n=11) | r = -0.13,  p = 0.65 | r = -0.13,  p = 0.30 | r = -0.70,  p = 0.01 ***** | r = -0.33,  p = 0.29 | r = -0.39,  p = 0.17 |
| **IL-18 (pg/mL)**  **Week 0**  (n=12) | r = 0.39,  p = 0.17 | r = 0.11,  p = 0.73 | r = 0.25,  p = 0.49 | r = 0.62,  p = 0.03 ***** | r = 0.17,  p = 0.56 |
| **IL-18 (pg/mL)**  **Week 24**  (n=11) | r = 0.53,  p = 0.10 | r = 0.22,  p = 0.58 | r = 0.67,  p = 0.08 | r = 0.72,  p = 0.02 ***** | r = 0.57,  p = 0.07 |

**Soluble markers are shown where significant correlations were observed at any time point.**

* Statistically significant (P value <0.05) using Spearman's rank sum test.
