# Supplementary figures and images for: Transient elastography score is elevated during rheumatoid factor-positive chronic hepatitis C virus infection and rheumatoid factor decline is highly variable over the course of direct-acting antiviral therapy
Source: PLoS One. 2022 Apr 28;17(4):e0267512. doi: 10.1371/journal.pone.0267512 (PMC9049346; doi:10.1371/journal.pone.0267512)

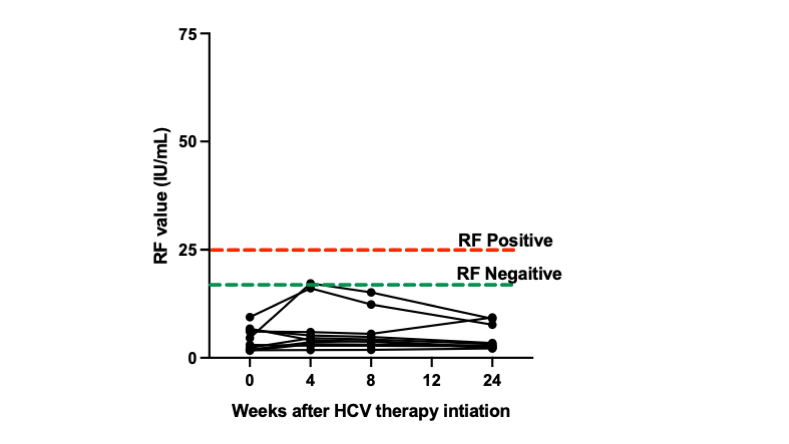

Supplement: S1 Fig — HCV infected (HCV+) RF negative (RF-; n = 10, <16 IU/mL) persons were followed longitudinally over the course of HCV DAA therapy and the serum RF values determined before (week = 0: n = 10), during (week 4: RF+ n = 0/10, 0%; HCV+ n = 0/10, 0%), (week 8: RF+ n = 0/10, 0%; HCV+ n = 0/10, 0%) and after (week 24: RF+ n = 0/10, 0%; HCV+ n = 0/10, 0%) treatment, where the proportion of the persons that were RF+ (>24 IU/mL) and had detectable serum HCV levels was determined. Differences between timepoints were determined by the paired Wilcoxon test. (TIFF) [file pone.0267512.s004.tiff]

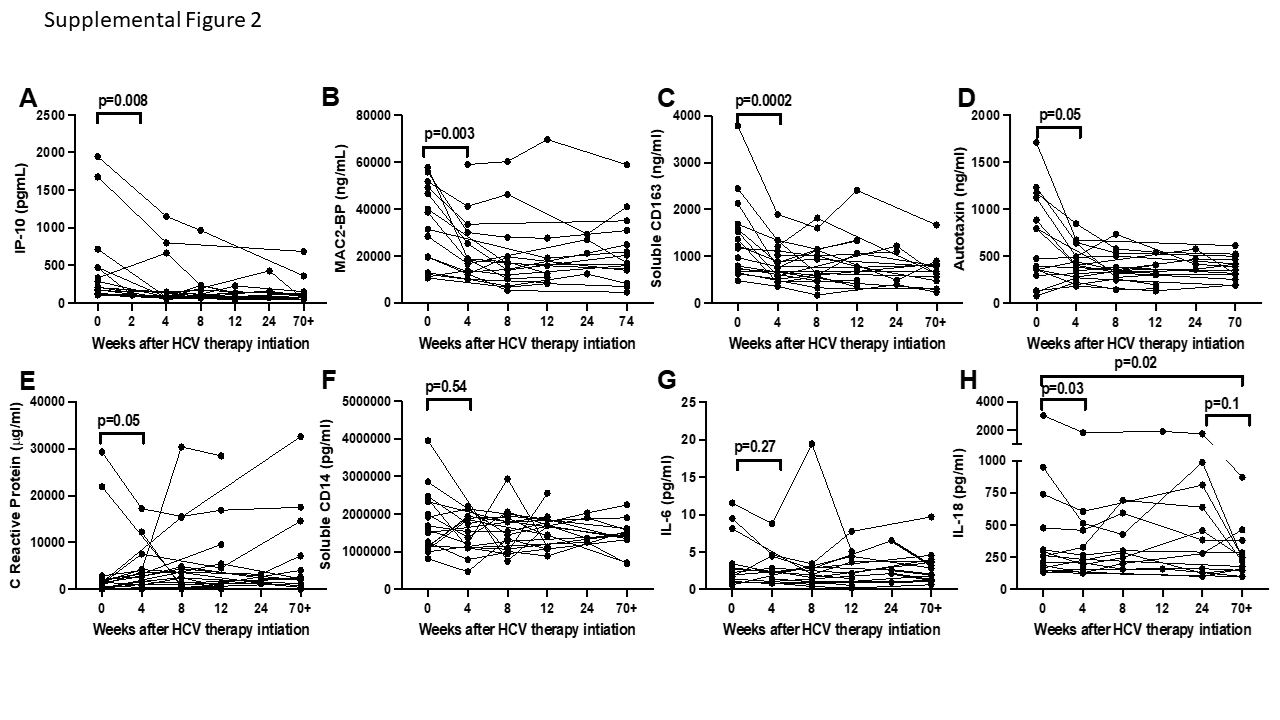

Supplement: S2 Fig — The plasma levels of systemic markers of immune activation including A) IP-10 B) MAC2-BP C) sCD163 D) autotaxin D) CRP E) sCD14 F) IL-6 and IL-18 were determined by ELISA at time points before (week 0), during (weeks 4, 8 and 12) and after (week 24) DAA therapy. Differences between time points were determined by the paired Wilcoxon test. (TIF) [file pone.0267512.s005.TIF]
